# Brain mechanisms of eye contact during verbal communication predict autistic traits in neurotypical individuals

**DOI:** 10.1038/s41598-020-71547-0

**Published:** 2020-09-03

**Authors:** Jing Jiang, Katharina von Kriegstein, Jiefeng Jiang

**Affiliations:** 1 Department of Psychiatry and Behavioral Sciences, Stanford University, Stanford, CA 94305 USA; 2 Max Planck Institute for Human Cognitive and Brain Sciences, 04103 Leipzig, Germany; 3 Berlin School of Mind and Brain, Humboldt-Universität zu Berlin, 10117 Berlin, Germany; 4 Institute of Psychology, Humboldt-Universität zu Berlin, 12489 Berlin, Germany; 5 Faculty of Psychology, Technische Universität Dresden, 01187 Dresden, Germany; 6 Department of Psychological and Brain Sciences, University of Iowa, Iowa City, IA 52242 USA

**Keywords:** Social behaviour, Human behaviour

## Abstract

Atypical eye contact in communication is a common characteristic in autism spectrum disorders. Autistic traits vary along a continuum extending into the neurotypical population. The relation between autistic traits and brain mechanisms underlying spontaneous eye contact during verbal communication remains unexplored. Here, we used simultaneous functional magnetic resonance imaging and eye tracking to investigate this relation in neurotypical people within a naturalistic verbal context. Using multiple regression analyses, we found that brain response in the posterior superior temporal sulcus (pSTS) and its connectivity with the fusiform face area (FFA) during eye contact with a speaker predicted the level of autistic traits measured by Autism-spectrum Quotient (AQ). Further analyses for different AQ subclusters revealed that these two predictors were negatively associated with attention to detail. The relation between FFA–pSTS connectivity and the attention to detail ability was mediated by individuals’ looking preferences for speaker’s eyes. This study identified the role of an individual eye contact pattern in the relation between brain mechanisms underlying natural eye contact during verbal communication and autistic traits in neurotypical people. The findings may help to increase our understanding of the mechanisms of atypical eye contact behavior during natural communication.

## Introduction

Eye contact is an important non-verbal component during social interaction (for reviews, see Refs.^1–3^). It occurs spontaneously and frequently during face-to-face communication^4,5^. An atypical pattern of eye contact is often observed in people with autism spectrum^6^ and atypical pattern of eye contact is also part of the criteria for diagnosing autism spectrum disorder (ASD)^7^. Autistic traits do not exist solely within the clinical population, but also in the general population^7–9^. Autistic traits are associated with particular eye contact behavior^10,11^. However, the brain mechanisms that link autistic traits and a particular eye contact pattern are unknown.

A network of brain regions is involved in eye contact processing in non-verbal situations (for reviews, see Refs.^1,2^) as well as in verbal communication^12^. The fast-track modulator model^2^ of eye contact has been developed mainly on findings that investigated eye contact in non-verbal situations. It assumes that this network is composed of subcortical [including superior colliculus (SC), pulvinar (Pulv) and amygdala (Amy)] and cortical visual areas [including lateral occipital cortex (LOC) and inferior temporal cortex (ITC)] interacting with brain regions of the so-called ‘social brain network’ [including Amy and orbitofrontal cortex (OFC) for emotion, pSTS and medial prefrontal cortex (mPFC) for intentionality, right anterior STS (aSTS) for gaze direction, and fusiform gyrus (FG) for face identity]. The regions within the social brain network are thought to interact with each other and to be modulated by dorsolateral prefrontal cortex (dlPFC). For verbal communication, a similar network has been identified in our previous study^12^. It includes increased responses in the right pSTS, left mPFC, the right dlPFC as well as visual cortices, and extensive enhanced connectivity between these regions and all other regions involved in the fast-track modulator model^2^. Several recent neuroimaging studies have shown that responses in part of this eye contact network, for example in the right pSTS, were associated with the amount of autistic traits in neurotypical individuals^13–15^. This relation was found in non-verbal situations in which participants passively watched faces with a direct or averted gaze. It remains unexplored whether a similar relation is also present between autistic traits and any regions and/or connectivity in the network for eye contact processing during verbal communication we identified in our previous study^12^. Addressing this question is important, because atypical eye contact is especially prominent in natural, complex, and cognitively demanding situations (e.g., when watching a speaker talking) in both ASD^16–20^ and neurotypical people with high autistic traits^21^.

Furthermore, neurotypical people show large inter-individual differences in looking preference for the eyes in both audiovisual speech perception^22^ and static face viewing^23,24^. These individual differences in looking behavior are very stable over time^23,24^. They have been associated with the level of overall autistic traits (Refs.^11,25^ but also see^26^) or subclusters (“Social” and “Attention-to-detail”) of autistic traits^10,27^. In addition, findings from neuroimaging studies in neurotypical^28^ and autistic^29^ individuals both suggested that variation in eye movement pattern influences brain responses. Therefore, it is currently unclear whether the association between brain responses and autistic traits found in previous studies^13–15^ are due to different types and amounts of visual input caused by individuals’ looking preferences or a processing mechanism itself that is independent of visual input. To clarify this, it is crucial to distinguish the brain mechanisms of eye contact (when participants are looking at the eyes) from those of looking at other facial features. It is also of great importance to consider the level of each participant’s looking preference for the eyes when investigating the relation between brain mechanisms of eye contact and autistic traits. It is likely that the brain mechanisms of eye contact are related to individual looking preference, which is in turn related to the overall and/or a specific subcluster of autistic traits.

Therefore, we had two goals in our study: First, to investigate whether the brain mechanisms underlying spontaneous eye contact during verbal communication are related to the overall and/or specific aspects of autistic traits in neurotypical individuals. Second, to examine whether an individual’s looking preference for the eyes mediates the relation between brain mechanisms of eye contact and the overall and/or specific aspects of autistic traits.

To address our goals, we conducted novel analyses based on fMRI data collected in our previous study^12^ and combined them with measures of AQ in the same subject group. In the fMRI study we created a naturalistic situation in which the listener made spontaneous eye contact while listening and watching another person talking. To do that, we presented participants with pre-recorded videos of speakers directly gazing at the camera while talking about daily life topics. The participants were instructed to listen to the speakers carefully and were allowed to freely look at different regions of the speaker’s face, as they would in daily communication. We simultaneously recorded fMRI and eye tracking data from the participants and used the fixations obtained from eye tracking data to define events for the fMRI analyses. This so-called fixation-based event-related fMRI (FIBER fMRI)^30,31^ allowed us to separate brain response and connectivity when participants spontaneously looked at the speaker’s eyes (Eyes events), from when they looked at the speaker’s mouth or elsewhere (Mouth or Off events)^12^.

We used the AQ^8^ to assess individual’s autistic traits. The AQ has good internal consistency^32^, predictive validity^33^, and test–retest reliability^34^ across different cultures in both people with ASD and the neurotypical population^9,35,36^. Previous studies have shown that factor analyses of the AQ resulted in two to five factors^9,37–39^. All studies reported two key factors/subclusters (“Social” and “Attention-to-detail”). These two factors have also been found to reliably and validly capture individual difference in autistic traits in a study with nearly a thousand people^9^. Important in the context of the present study, these subclusters are associated with individual differences in face recognition in the general population^10,40,41^. Therefore, we adopted the two-factor/subcluster classification of AQ to examine whether the two different subclusters of autistic traits as measured by AQ are related to neural mechanisms of eye contact. We used multiple regression analyses to address whether brain response and effective connectivity during eye contact as compared to mouth fixation could predict an individual’s AQ total score and/or AQ subcluster score^7^. We then used mediation analyses to examine whether participants’ looking preferences to the speaker’s eyes could mediate the relation between brain mechanisms for eye contact and the AQ total scores and/or AQ subcluster scores.

## Results

### BOLD response and effective connectivity during eye contact predicted autistic traits

**Figure 1:**
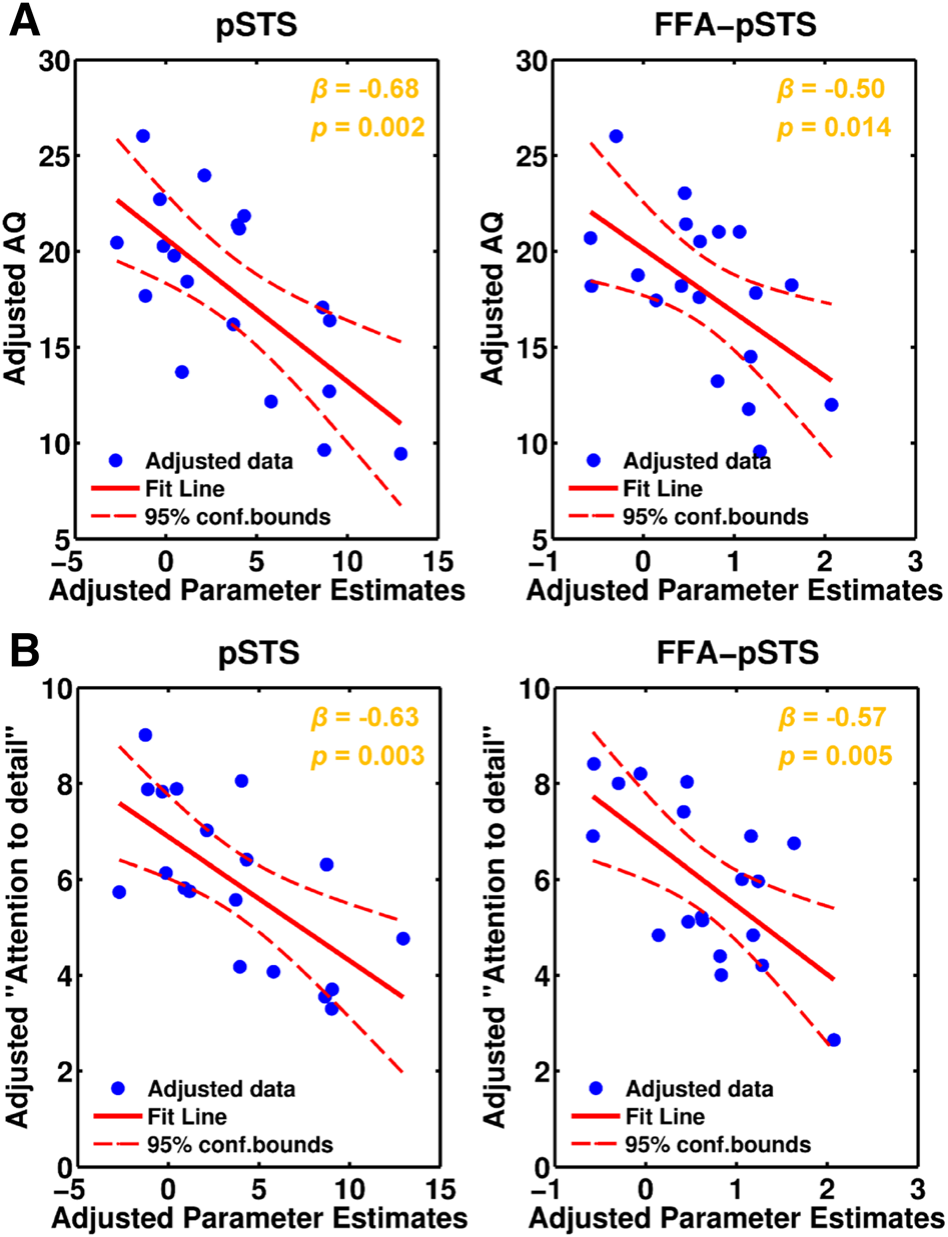
Partial regression scatter plots for BOLD response and connectivity predicting autistic traits. (**A**) Partial regression plots for the AQ total scores. (**B**) Partial regression plots for the “Attention to detail” subcluster scores. Significant predictors for both AQ total scores and “Attention to detail” subcluster scores were the BOLD response in the pSTS and the effective connectivity of FFA–pSTS in the Eyes vs. Mouth contrast.

To address our first goal, whether changes in the brain response and/or effective connectivity predict variation in autistic traits, we conducted a multiple linear regression (MLR) analysis using the stepwise method (*p* < 0.05 to enter, *p* > 0.1 to remove). Parameter estimates (referring to beta weights) extracted from both brain regions that showed increased BOLD response and enhanced effective connectivity in PPI analysis in Eyes vs. Mouth contrast identified in our previous study^12^ (for details see “Methods” section) were entered as independent variables (IVs) and the AQ total scores as dependent variable (DV) (Table 1). For AQ total scores we identified 2 significant predictors (adjusted R^2^ = 0.47, *F* (2, 16) = 8.87, *p* = 0.003, Cohen's f^2^ = 1.13) (Table 2): the brain response in pSTS (*β* =  − 0.68, *t* =  − 3.79, *p* = 0.002) and FFA–pSTS effective connectivity (*β* = − 0.50, *t* = − 2.77, *p* = 0.014) in the Eyes vs. Mouth contrast. These 2 predictors showed significant negative correlation with the AQ total scores across participants. Namely, decreased pSTS response and reduced FFA–pSTS effective connectivity predicted higher autistic trait scores (Fig. 1A). The collinearity statistics showed that the tolerance was 0.93 and the variance inflation factor (VIF) was 1.08 for all the predictors, indicating no multicollinearity between the predictors.

**Table 1:** AQ scores, subscale scores and subcluster scores for each participant.

Subjects	AQ	Communication	Social skills	Imagination	Attention switching	Attention to detail	Social subcluster
sub01	21	3	3	2	6	7	14
sub02	12	0	1	2	4	5	7
sub03	20	2	2	2	5	9	11
sub04	10	0	0	3	5	2	8
sub05	9	0	3	0	0	6	3
sub06	20	3	1	4	6	6	14
sub07	19	4	0	4	4	7	12
sub08	21	5	2	5	5	4	17
sub09	12	0	1	1	4	6	6
sub10	26	7	1	2	8	8	18
sub11	21	3	3	3	6	6	15
sub12	17	3	2	5	3	4	13
sub13	21	2	4	3	5	7	14
sub14	20	3	1	3	5	8	12
sub15	12	1	2	3	2	4	8
sub16	21	1	5	0	9	6	15
sub17	15	4	2	1	3	5	10
sub18	18	4	5	2	3	4	14
sub19	26	3	3	4	7	9	17
Mean	17.95	2.53	2.16	2.58	4.74	5.95	12
SD	4.99	1.90	1.46	1.46	2.10	1.87	4.08

**Table 2:** Stepwise multiple linear regression results for AQ and AQ subcluster.

Dependent variables (DV)	Significant predictors	Coefficients				Significance test of model			
		Unstandardized B	Standardized Beta	t	Sig	R^2^	Adjusted R^2^	F	Sig
AQ	pSTS	− 0.75	− 0.68	− 3.79	0.002	0.53	0.47	8.87	0.003
	FFA–pSTS	− 3.32	− 0.50	− 2.77	0.014				
Attention to detail	pSTS	− 0.26	− 0.63	− 3.53	0.003	0.53	0.47	9.09	0.004^a^
	FFA–pSTS	− 1.44	− 0.57	− 3.24	0.005				

^a^p value after Bonferroni correction (n = 2).

To check which subcluster of autistic traits can be specifically predicted by the brain response and/or effective connectivity identified for overall autistic traits, we also conducted MLR analysis for each AQ subcluster separately. We used the significant predictors (pSTS response and FFA–pSTS effective connectivity) identified for AQ total scores as IVs, the scores of “Social” or “Attention to detail” subcluster as the separate DV. We found these 2 predictors specifically predicted the scores on the “Attention to detail” subcluster (adjusted R^2^ = 0.47, *F* (2, 16) = 9.09, *p* = 0.004 after Bonferroni correction (n = 2), Cohen's f^2^ = 1.13) (Table 2). The pSTS response (*β* =  − 0.63, *t* = -3.53, *p* = 0.003) and FFA–pSTS effective connectivity (*β* =  − 0.57, *t* =  − 3.24, *p* = 0.005) in the Eyes vs. Mouth contrast both showed a significant negative correlation with the “Attention to detail” subcluster scores (Fig. 1B). There was no multicollinearity between the predictors (tolerance = 0.93, VIF = 1.08). We did not find any significant predictor for the “Social” subcluster. In the following text, we refer to this set of analyses and results for AQ total scores and AQ subcluster scores as MLR I.

### Looking preference for eyes mediated the relation between brain mechanisms of eye contact and “Attention to detail” aspects of autistic traits

**Figure 2:**
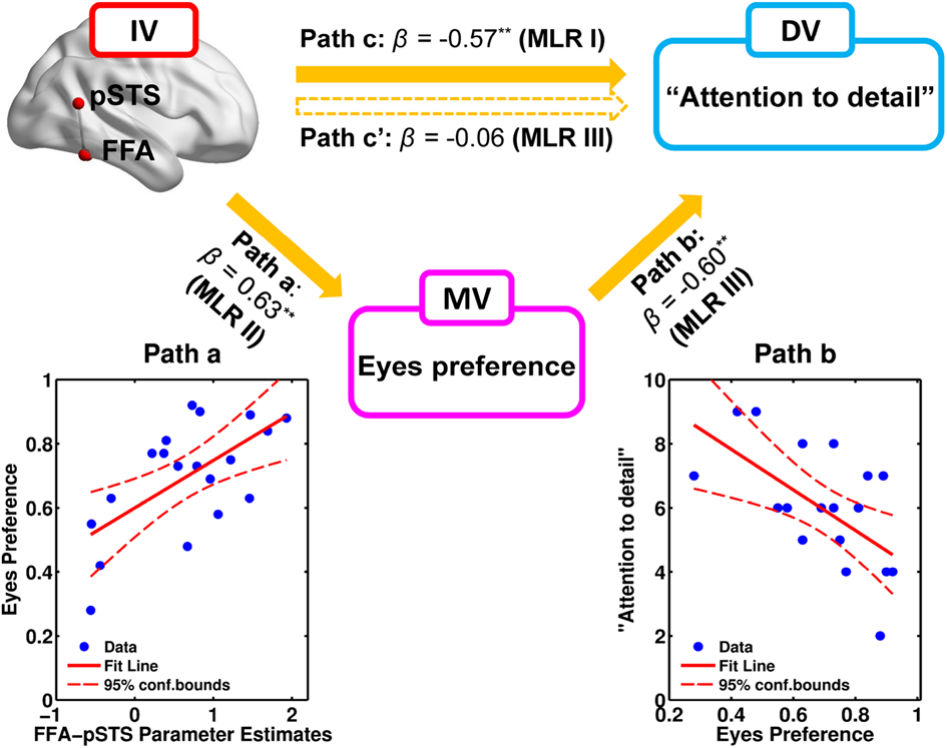
Mediation role of looking preference for eyes (MV) on the relation between the FFA–pSTS connectivity (IV) and the “Attention to detail” subcluster (DV). Path c showed the direct correlation between the IV and the DV when considered alone. Path c′ showed the correlation between the IV and the DV when added with MV. The paths a and b together indicated that the IV predicted the DV through the mediation of MV.

To address our second goal, whether the looking preference for the eyes of speakers (mediator variable, MV) mediates the relation between brain response/connectivity during eye contact (IV) and AQ scores (DV), we conducted mediation analyses by following procedures recommended by Baron and Kenny^42^ (see “Methods” section). First, as shown in the MLR I presented above, the IVs (the pSTS response and FFA–pSTS effective connectivity) were significantly related to the DV (the overall autistic traits and “Attention to detail” subcluster of autistic traits), meeting the first criterion (Fig. 1, Table 2). Second, in MLR II, we found that one IV (the FFA–pSTS connectivity) was significantly and positively related to the DV (MV: Eyes Preference) (adjusted R^2^ = 0.36, *F* (1, 17) = 11.16, *p* = 0.004; *β* = 0.63, *t* = 3.34, *p* = 0.004) (Fig. 2, path a), meeting the second criterion. Third, in MLR III, we found (i) the MV (Eyes Preference) was only significantly related to the “Attention to detail” subcluster (adjusted R^2^ = 0.32, *F* (1, 17) = 9.33, *p* = 0.007; *β* =  − 0.60, *t* =  − 3.06, *p* = 0.007) (Fig. 2, path b). (ii) The initial significant correlation between the IV (the FFA–pSTS effective connectivity) and DV (the “Attention to detail” subcluster scores) (*β* =  − 0.57, *t* =  − 3.24, *p* = 0.005) (Fig. 2, path c) in the MLR I was reduced and became insignificant (*β* =  − 0.06, *t* =  − 0.21, *p* = 0.835) (Fig. 2, path c′), meeting the third criterion for the mediation effect by looking preference for the eyes.

The mediation effect was also confirmed by an additional analysis using Sobel test (see Supplementary Materials). Taken together, these results indicated that the looking preference for the eyes was a full mediator in the relation between FFA–pSTS effective connectivity and the “Attention to detail” subcluster during eye contact.

## Discussion

In this study, we recorded individuals’ spontaneous eye contact in the scanner and investigated the relation between the brain mechanisms of natural eye contact during verbal communication and autistic traits in neurotypical individuals, and the role of individual eye contact preference in this relation. We found that the brain response in the pSTS and the effective connectivity between FFA and pSTS during eye contact in contrast to mouth fixation were negatively associated with the AQ total scores and specifically the “Attention to detail” subcluster scores. In addition, the relation between the FFA–pSTS connectivity and the “Attention to detail” subcluster scores was mediated via the looking preference for the speaker’s eyes. These results provide first evidence that part of the brain mechanisms during spontaneous eye contact when watching a speaker talking is related to the overall and especially to the attention to detail aspects of autistic traits. In addition, the individual eye contact preference mediates this relation.

We found the relation between response in the right pSTS during spontaneous eye contact and the AQ total scores, especially the “Attention to detail” subcluster scores, in a verbal context. Nummenmaa et al.^13^ found neurotypical individuals’ AQ scores positively correlated with response in the right pSTS when viewing faces with variable gaze, but negatively correlated with that when viewing constant gaze. In another study, both white matter volume and BOLD response to a stroop task in the pSTS were negatively correlated with the AQ scores^15^. Interestingly, Nummenmaa et al.^13^ and von dem Hagen et al.^15^ also found response and white matter volume in the pSTS were negatively related to “Attention to detail” subcluster scores. Consistent with these findings, our study showed that reduced pSTS response when viewing speakers with constant gaze is associated with increased AQ total scores and specifically “Attention to detail” subcluster scores. This indicates that the overall and especially the attention to detail aspects of autistic traits can be predicted by the pSTS response during eye contact in both nonverbal and verbal communication situations. Importantly, the contrast (Eyes vs. Mouth events) in our study was controlled for the visual input type by recording participants’ natural looking behaviors. In addition, the association between AQ scores and “Attention to detail” scores and pSTS response was not mediated by the individual looking preference for eyes. Thus, this association shown in our study is unlikely due to the different amount of visual input caused by individual’s eye contact preference that may correlate with autistic traits. Instead, it is probably due to different brain mechanisms per se used by neurotypical people with different levels of autistic traits during eye contact while watching a speaker talking.

Besides the pSTS response, we additionally found a negative correlation between the FFA–pSTS connectivity during spontaneous eye contact and the AQ scores, and specifically “Attention to detail” subcluster scores. Correlations between AQ scores and brain connectivity were not investigated in previous studies^13–15^. Furthermore, the looking preference for the eyes mediated the relation between the FFA–pSTS connectivity and the “Attention to detail” subcluster scores. We found individuals’ looking preference for the speaker’s eyes positively correlated with the FFA–pSTS connectivity in the brain. Currently, there is an ongoing debate about the interaction between the FFA and pSTS. On one hand, the FFA and pSTS are two main brain regions specialized for face perception^43,44^. The FFA is considered to be involved more in processing relatively invariant aspects of the face, such as identity^43–46^, while the pSTS is thought be important in processing dynamic changes in the face, such as gaze, expression, and facial movement^44,47–49^. Thus, these two regions have been regarded as working independently in parallel pathways, although they respond simultaneously in face processing^44^. On the other hand, FFA–pSTS connectivity has been found when participants viewed eye gaze shifted towards them as compared to away from them^50^ and when they perceived changes in facial expression and gaze on faces with same identity compared with those with different identities^51^. The FFA and pSTS are also intrinsically connected in functional resting state^52^. In the present study, the FFA–pSTS connectivity was obtained in the Eyes vs. Mouth contrast in an emotionally neutral context. The peak coordinate of pSTS (x = 51, y =  − 48, z = 9) is very close (distance ca. 7 mm in total) to that of pSTS for gaze processing found in previous studies^49,53^. Thus, the interaction between FFA and pSTS might be due to the integration of gaze information and identity or more invariant face information. The pSTS has also been proposed to serve a role in mentalizing, that is, inferring another person’s mental state or intentions^2,54–56^. It seems that connectivity between the FFA and pSTS could also indicate a process of inferring information from the eyes about the speaker’s intention. However, the coordinate of pSTS revealed in previous meta-analyses tends to be more dorsal^57,58^ in contrast to that of the current study. We therefore speculate that it is more likely that the FFA–pSTS connectivity represents the integration of gaze and identity information.

We also found that the looking preference for the eyes was negatively correlated with the “Attention to detail” subcluster scores. This seems to be contrary to a previous study which found that looking at the eyes more was associated with higher scores in “Attention to detail” in a face identity learning task^10^. We attribute this discrepancy to specific tasks used in the two studies. During face identity learning, eye region is considered as the most important detail on the face (for reviews, see Refs.^1,59,60^). Stronger looking preference for the eyes in people with higher “Attention to detail” scores in such a task therefore seems reasonable^10^. However, in our study participants performed a speech recognition task. In this situation, non-eye regions (i.e., orofacial movement) usually provide more details for speech recognition than the eye region do^22,61,62^. This might be the reason we find a negative correlation between the Eyes preference and the “Attention to detail” subcluster scores.

The “Social” subcluster scores were not predicted by any brain response or effective connectivity during eye contact. von dem Hagen et al.^15^ found that white matter volume and BOLD response to a stroop task in the pSTS was negatively correlated with “Social” subcluster scores. Yet, no study has reported any brain response or connectivity to eye gaze was related to “Social” subcluster scores. The “Social” subcluster scores in our study were also not related to the individual looking preference for eyes. One possible reason is that the pre-recorded videos used in the current study did not provide enough social information. Recent studies showed that neurotypical participants looked significantly less at a live person (or a pre-recorded person they believed to be “live”) than a pre-recorded person (Refs.^21,63^, but see also Ref.^25^). In live interaction, individuals with higher autistic traits looked significantly less at the experimenter (Ref.^21^, but see also Ref.^25^). Participants’ social skills scores measured by AQ correlated with the looking preference towards live but not videotaped persons^63^. However, whether looking preference is always correlated with social skills or subclusters in live interactions is unclear in other studies^21,25^, as they did not investigate different subscales or subcluster of the AQ. Thus, it is currently an open question whether the lack of correlations between the “Social” subcluster scores and brain response/connectivity and eye contact pattern are due to the pre-recorded nature of the videos used in our experimental paradigm.

An alternative explanation could be the lack of variance of the “Social” subcluster scores. There was little variation between participants on 3 of the 4 social subscales, namely “social skills,” “communication,” and “imagination,” except “attention switching” (Table 1). In contrast, scores on “Attention to detail” varied more between participants. These results are in agreement with previous studies on large samples (n > 1,000) of neurotypical participants regardless of culture^8,35^. We therefore performed a supplementary MLR analysis for each subscale in the “Social” subcluster (see Supplementary Materials) and found that both pSTS response and FFA–pSTS connectivity predicted “attention switching” scores, in which there is larger variation between participants (0–9), but not for the other 3 subscales with little variation between participants. Thus, the lack of correlation between the social subcluster and brain response or connectivity might be due to the lack of variance in the “Social” subcluster scores measured by AQ. Another possibility is that pSTS response and FFA–pSTS connectivity to eye contact may be particularly relevant for attention-related aspects of autistic traits.

Individuals with ASD have been repeatedly reported to show less pSTS response to eye gaze in non-verbal contexts (e.g. Refs.^64,65^), and autism severity was negatively related to pSTS response^64^. These findings are similar to ours that strong autistic traits were associated with reduced pSTS response, albeit in a verbal context and in neurotypical participants. Whether these similar findings represent the same underlying brain mechanisms is an open question. Our imaging findings are based on eye tracking data, that is, response and connectivity when participants are looking at the eye region spontaneously, whereas previous studies on ASD did not consider this important factor (e.g. Refs.^64,65^). Furthermore, there are large individual differences in both eye gaze pattern^6^ and autistic traits (e.g. social aspects) in people with ASD (Ref.^66^ for a review see^67^). Thus, individualized eye gaze pattern in ASD may also relates to different aspects of autistic traits as neurotypical people in the present study. Unfortunately, none of the previous studies on eye contact in ASD have examined subclusters of autistic traits. Therefore, including people across the whole autism spectrum in the same context and considering the effect of the individual eye contact pattern and the subcluster of autistic traits, will yield a better understanding of the relationship between autistic traits, eye contact pattern, and brain mechanisms of eye contact in face-to-face communication.

A limitation of the present study is its relatively small sample size. Increasing sample size is difficult in studies with a complex paradigm, e.g., due to the fact of difficulty in obtaining complete and valid eye tracking data for every recruited participant in the scanner (see “Participants” in “Methods” section). Small sample sizes are not ideal when testing individual differences on autistic traits, though the subclusters of autistic traits showed similar patterns to those in studies with large sample size as discussed above. The effect of small sample size on replicability of studies is controversially discussed (see^68^ but also^69^). In our study, we have a very large effect size indicated by cohen’s f^2^ = 1.3 and R^2^ = 0.53 in both MLR I and II. The effect size together with the desired probability level 0.05, the number of predictors = 2 in the present MLR models, and the desired statistical power level = 0.8, the minimum required sample size is 11 via G*Power analysis^70^. Our sample is larger than the minimum required size.

Previous studies reported that intelligence quotient (IQ) relates to eye gaze processing (e.g., Peterson and Miller^71^) in an emotional context. In the present study we did not acquire measures of intelligence. It would be interesting to test whether the IQ is also associated with brain mechanisms involved in eye gaze processing in a non-emotion context, such as the verbal communication in the present study.

In summary, using an ecologically valid paradigm, our study presents initial evidence that pSTS response and its connectivity to the FFA are related to autistic traits in neurotypical individuals and that part of this relation is mediated by the individual eye contact preference. The present study is the first to show that this relation holds in a verbal communication context and that it is also present when controlling for the potential different sensory input due to the different spontaneous eye contact patterns related to autistic traits. In addition, this study demonstrates a link between brain mechanisms underlying eye contact and a subcluster of autistic traits in neurotypical people. Evidence emerges that “Social” and “Attention to detail” subclusters of autistic traits are differently associated with brain structures in both neurotypical people^15^ and people with ASD^72^. Thus, the present study also highlights the importance of studying different subcluster aspects of autistic traits when exploring the neurobiology of autistic traits, rather than only the overall autistic traits.

## Methods

### Participants

Thirty healthy native German adults (15 female, 15 male; 27.5 years old ± 3.6 SD) participated in the experiment. They reported no history of psychiatric or neurological disease. All were right handers^73^ with normal vision (without correction). All participants provided written informed consent. The study protocol and all methods performed were approved by the Research Ethics Committee of the University of Leipzig (AZ: 192-14-14042014) and in accordance with its relevant guidelines and regulations. Nine participants were excluded due to difficulties obtaining eye tracking data (e.g. difficulties with calibration before the experiment or eye tracking during the experiment). Another two participants were excluded because of excessive head movement (> 3 mm) in the MRI scanner. Thus, eye tracking and fMRI data analyses were based on 19 subjects (11 female, 8 male; 26.0 ± 2.6 SD year-old).

### Autistic traits

To measure autistic traits, all participants filled in a German translation of AQ^8^ provided by Freitag et al.^74^. The AQ is a widely used questionnaire to quickly and easily measure variation in autistic traits in both clinical and general populations^8^. It consists of 5 subscales. The subscales of AQ were validated in studies with large population samples, e.g., the original paper reporting design of AQ by Baron-Cohen et al.^8^ (see “Item Analysis and Internal Consistency”), the revalidation paper by Hoekstra et al.^9^ (see “Internal Consistency and Test–Retest Reliability”). We classified the 5 subscales into 2 subclusters as in most recent studies (e.g. Refs.^9,10,75^): “Social” subcluster (combining “communication [items 7, 17, 18, 26, 27, 31, 33, 35, 38, 39],” “social skills [items 1, 11, 13, 15, 22, 36, 44, 45, 47, 48],” “imagination [items 3, 8, 14, 20, 21, 24, 40, 41, 42, 50],” and “attention switching [items 2, 4, 10, 16, 25, 32, 34, 37, 43, 46]” subscales; higher scores indicate higher likelihood of deficit in social ability) and “Attention to detail” subcluster (referring to the “attention to detail [items 5, 6, 9, 12, 19, 23, 28, 29, 30, 49]” subscale; higher scores indicate higher likelihood of exceptional in attention to detail ability).

Each subscale contains 10 items. The participants rated to what extent they agree or disagree with the items on a 4-point Likert scale (“definitely agree,’’ ‘‘slightly agree,’’ ‘‘slightly disagree,’’ and ‘‘definitely disagree’’). Each item scores 1 point if the response represents autistic-like behavior (e.g. exceptional attention to detail, poor attention switching). The AQ total scores and scores in each subcluster were summed separately. The AQ total score for each participant was below the cut-off value (32) that is indicative of a manifestation of autistic traits typical for ASD (17.95 ± 4.99SD) (Table 1). The test of normality (Shapiro–Wilk test, *p* > 0.05, n = 19) showed that both the AQ total scores and subcluster scores were distributed normally.

### Stimuli

The stimuli were eight ca 6-min long monologue videos from 4 German speakers (for details see Supplementary Materials).

### Experimental procedures

The fMRI experiment consisted of 4 sessions implemented in Presentation software (version 14.5, Neurobehavioral Systems Inc., USA). Each session had one Normal and one Noise video from speakers of different sexes. The orders of speakers’ sexes and video conditions were counterbalanced across sessions and participants. We instructed the participants to carefully watch and listen to the speaker talking, they were allowed to freely look at different regions of the speaker’s face. At specific time points the videos were stopped, participants performed a speech recognition task by answering “What was the last word you heard?” from 3 choices shown on the screen. They chose the answer by pressing one of 3 corresponding buttons on a response-box. The video continued when a button was pressed or after 4 s without a response.

### Eye tracking

We used a 120 Hz monocular MR-compatible eye tracker (EyeTrac 6, ASL, USA) to record participants’ eye movements during the experiment. Prior to the fMRI experiment, the eye tracking system was calibrated using a standard nine-point calibration procedure for each participant. Before each session, the accuracy of eye tracking was checked. If necessary, the eye tracking system was recalibrated.

### Imaging data acquisition

Functional images and structural T1-weighted images were obtained using a 3T Siemens Tim Trio MR scanner (Siemens Healthcare, Erlangen, Germany), equipped with a 12-channel head coil. For other scanning details see Supplementary Materials.

### Eye tracking analysis

#### Fixation events for fMRI analyses

We used EyeNal software (ASL, USA) and customized MATLAB scripts for the eye tracking data analysis. A fixation was defined as having a minimum duration of 100 ms and a maximum visual angle change of 1 degree. Natural speaking is often accompanied by head movements. We therefore corrected the position of participants’ fixations based on the speakers’ head movements in the videos using the Tracker software (https://www.cabrillo.edu/~dbrown/tracker/) (for details see Supplementary Materials). We labeled fixations within the areas of interest (AOIs) of eyes and mouth as “Eyes” and “Mouth” respectively, and fixations outside these AOIs as “Off” (For details, see Ref.^12^). Fixations occurring consecutively within the same AOI were concatenated into one fixation, resulting in one event for the fMRI analyses. The event onset was the start time of the fixation forming in the corresponding AOI.

#### Eye gaze patterns

As reported previously^12^, the eye gaze patterns made this experiment suitable for fMRI analysis as a rapid event-related design: There was a sufficient number of events (NE, Fig. S1A, Table S2) and suitably long for inter-event intervals (IEI, Fig. S1B, Table S3) the Eyes and Mouth events across participants. Both indices (NE and IEI) were roughly balanced between event types across conditions (Fig. S1A,B). In addition, the IEI was jittered (Fig. S1C) and the events occurred in a variable order (Fig. S1D).

#### Looking preference for the eyes

To define each participant’s looking preference for a speaker’s eyes, we computed an Eyes Preference index defined as N_eyes_/N_non-eyes_. N_eyes_ is the number of Eyes events, N_non-eyes_ is the sum number Mouth and Off events. A larger value means a stronger looking preference for the eyes.

### fMRI analyses

In the present study, for brain response we used the results reported in our previous study^12^. For brain connectivity, we reanalyzed with a similar region of interest (ROI)-based psychophysiological interaction (PPI) analyses as reported previously^12^. However, in contrast to the previous analyses we added head movement parameter that was neglected in Jiang et al.^12^.

#### Analyses of BOLD response

We performed all fMRI analyses using Statistical Parametric Mapping software (SPM8, Wellcome Trust Centre for Neuroimaging, UCL, UK, https://www.fil.ion.ucl.ac.uk/spm) (see Supplementary Materials). The results for BOLD response showed four large clusters involved in eye contact (Eyes > Mouth) (Fig. S2): (1) bilateral visual cortices, including the cuneus (Cun, BA 17/18), calcarine sulcus (Cal, BA 17/18) covering V1, V2, and V3 and extending to the precuneus (Prec, BA 7), (2) the right temporal-parietal junction, including the angular gyrus and supramarginal gyrus and extending into the pSTS (TPJ&pSTS, BA 39/40) (Note that the pSTS involved in eye contact processing is more posterior to that involved in mouth movement processing, this is consistent with previous findings (e.g., Pelphrey et al.^64^; Pelphrey et al.^49^), (3) the left medial prefrontal cortex (mPFC), including the anterior cingulate cortex extending to medial orbital frontal cortex (BA 10/24/32), and (4) the right dorsolateral prefrontal cortex (dlPFC) (BA 9/46) (FWE cluster-wise corrected, *p* < 0.05). These results were reported in Jiang et al.^12^.

#### Analyses of effective connectivity

For the effective connectivity, we conducted ROI-based psychophysiological interaction (PPI) analyses^76^. PPIs have been considered simple models of effective connectivity^77^ (for details about analyses procedure, see Supplementary Materials). The ROIs were the same ROIs as reported in Jiang et al.^12^. The ROIs included (i) those regions that had significant BOLD response to eye contact (vs mouth fixation) in verbal communication (Jiang et al.^12^), and (ii) those regions that have been implicated in the predominant model for eye contact processing, i.e., the fast-track modulator model^2^. We used the ROIs that were responsive to eye contact (vs mouth fixation) in verbal communication because the main purpose of the present manuscript was to find predictors of autistic traits among regions specifically responsive to eye contact in verbal communication. We also used the ROIs that have been implicated in the fast-track modulator model^2^ for connectivity analyses, because all the specific connectivity proposed in this model was proposed based on evidence of eye contact processing in non-verbal contexts. It is unknown whether all the specific connectivity for non-verbal contexts would be also shown in the same way in verbal communication (see Jiang et al.^12^). Therefore, we defined these ROIs anatomically. Most of the regions are defined in the WFU_PickAtlas^78^ or the SPM Anatomy toolbox (v2.1)^79^. However, there were still a few regions not available in these toolboxes. For regions that were not available in the toolboxes, we defined via probabilistic maps (FFA and aSTS) or anatomically (SC) with reference to a brain atlas^80^. All regions were used as both source ROIs and target ROIs (for details see Supplementary Materials). Note that for regions with explicit hemispheric prediction in the fast-track modulator model or showing lateralized activation in the brain response analyses (e.g., right aSTS predicted in the model, right pSTS predicted in both model and showed significant lateralized activation in the response analysis, right dlPFC showed significant lateralized activation in the response analysis), we used the lateralized region as ROI. However, for regions with no explicit hemispheric prediction in this model, we also did not find significant laterality/hemispheric effects on activations between bilateral regions (p > 0.05 after FDR correction for all regions), indicating no strong functional dissimilarity between bilateral regions. Therefore, we merged bilateral regions as a single ROI to extract signal better reflecting general rather than lateralized information for connectivity analyses as done in previous studies (e.g., Admon et al.^81,82^). We conducted PPI analyses between all ROIs to identify all the possible connectivity that specific to eye contact in verbal communication. To obtain all potential contributors (connectivity) to accounting for the variances in autistic traits, we performed small-volume correction (FWE voxel-wise) for each target ROI in the PPI analyses. All the connectivity survived after correction was further used as independent variables for multiple linear regression analyses.

In contrast to the PPI analyses reported on the same data set in Jiang et al.^12^, here we additionally added a head movement parameter (framewise displacement, FD)^82^ to eliminate possible artefacts arising from head movement. After regressing out the head movement effect, the PPI results (Fig. S3) showed a very similar pattern as that in Jiang et al.^12^. It showed that the effective connectivity results we found for eye contact was relatively robust to the effect of head movement. The significant effective connectivity during eye contact as compared to mouth fixation was plotted in Fig. S3 (*p* < 0.05, FWE corrected).

### Multiple linear regression analyses

We conducted the multiple linear regression analyses in SPSS (Version 20.0, IBM Corp., USA). For IVs, we used the MarsBar toolbox^83^ to extract each participant’s parameter estimates from the individual’s Eyes vs. Mouth contrast file generated in the 1st level analyses for brain response and connectivity. The parameter estimates for the brain response were from brain regions showing increased BOLD response to the contrast eyes vs. mouth (i.e., 10-mm radius spheres centered on group statistical maximum coordinates. For details, see ‘Functionally defined ROIs’ in Supplementary Materials). The parameter estimates for brain connectivity were from target ROIs showing enhanced effective connectivity in the PPI analyses (For details about the definition, see ‘Definition for target ROIs’ in Supplementary Materials). The standardized *β* coefficients were used to evaluate the correlation level between the IVs and DV. Note that there was no significant difference between males and females on the AQ total scores (*t* =  − 0.67, *p* = 0.513) and no significant correlation between AQ and age (*r* = 0.11, *p* = 0.650). We therefore did not include sex or age as covariates.

### Mediation analyses

For mediation analyses, three criteria need to be met for the mediation effect in the following procedures recommended by Baron and Kenny^42^. First, IV must be significantly related to DV when considered alone. This was tested in the MLR I described above. Second, the IV must be significantly related to MV in an additional MLR analysis (MLR II) in which IV was the significant predictors identified in MLR I, and the MV was DV. Third, in the final MLR model (MLR III) in which both the significant predictors identified in MLR II and MV were entered as IVs, (i) the MV must be significantly correlated with the DV, and (ii) the correlation between the IV and DV would decrease in magnitude and significance from the level shown in the MLR I when the MV was not involved.

We repeated the same analysis for each AQ subcluster by entering AQ subcluster scores as separate DV. To check the validity of the results, we conducted an additional conservative test, namely the Sobel test^84^ (see Supplementary Materials).

## Supplementary information

Supplementary Information
