# Supplementary material for: Brain mechanisms of eye contact during verbal communication predict autistic traits in neurotypical individuals
Source: Sci Rep. 2020 Sep 3;10:14602. doi: 10.1038/s41598-020-71547-0 (PMC7471895; doi:10.1038/s41598-020-71547-0)
Supplement: Supplementary file 1 — Supplementary Information [file 41598_2020_71547_MOESM1_ESM.pdf]

Supplementary Materials

Title: Brain Mechanisms of Eye Contact during Verbal Communication Predict Autistic Traits in Neurotypical Individuals

Author names and affiliations:

Jing Jiang[1,2,3,4*], Katharina von Kriegstein[2,4,5], Jiefeng Jiang[6]

[1]Department of Psychiatry and Behavioral Sciences, Stanford University School of Medicine, Stanford, California 94305, USA

[2]Max Planck Institute for Human Cognitive and Brain Sciences, Leipzig, 04103, Germany

[3]Berlin School of Mind and Brain, Humboldt-Universität zu Berlin, Berlin, 10117, Germany

[4]Institute of Psychology, Humboldt-Universität zu Berlin, Berlin, 12489, Germany

[5]Faculty of Psychology, Technische Universität Dresden, Dresden 01187, Germany

[6]Department of Psychological and Brain Sciences, University of Iowa, Iowa City, Iowa 52240, USA

***Corresponding author:**

Jing Jiang

Department of Psychiatry and Behavioral Sciences

Wu Tsai Neurosciences Institute

Stanford University

401 Quarry Road

Stanford, CA 94305

Email: jingjiangpsy@gmail.com

Tel: +1 650 382 9582

Methods

Stimuli

The stimuli were 8 monologue videos from 4 German speakers (2 females and 2 males; 20, 22, 24, and 24 year-olds, respectively). Each monologue lasted approximately 6 mins and was about daily life topics, e.g., a description of a typical week or how to learn a foreign language. The speakers narrated these emotionally neutral topics in a natural and emotionally neutral manner. Eye contact is an event in which two partners look at each other's eyes at the same time (also called mutual gaze) [1]. We simulated such a situation by recording the front view of speakers with their gaze constantly directed towards a person behind the camera, to make the speakers feel as though were talking to a real person. For more recording details, see Jiang, et al. [2].

Our daily communications occur in either relatively quiet or noisy situations. Therefore, the videos were presented in two conditions: the original videos with high signal-to-noise ratio (SNR) (Normal condition) and videos with low SNR (Noise condition) by mixing the audio tracks of the videos with background noise (i.e., people talking and the clatter of dishes in a cafeteria). When watching and listening to a speaker, listeners fixate more on the eyes. However, if the speech signal is noisy, fixations on the mouth increase[3,4]. Thus, the Noise condition allowed us to obtain balanced fixations between eyes and mouth.

Imaging data acquisition

A gradient-echo EPI (echo planar imaging) sequence was used for the functional MRI (TE 30 ms, flip angle 90 degrees, TR 2.79 s, whole brain coverage with 42 slices, acquisition bandwidth 116 kHz, 2 mm slice thickness, 1 mm inter-slice gap, in-plane resolution 3 mm × 3 mm). Geometric distortions were characterized by a B0 field-map scan. The field-map scan consisted of gradient-echo readout (24 echoes, inter-echo time 0.95 ms) with standard 2D phase encoding. The B0 field was obtained by a linear fit to the unwrapped phases of all odd echoes. Before the functional runs, structural images were also acquired for each participant using a high-resolution, T1-weighted 3D MP-RAGE sequence: TI=650 ms, TR=1300 ms, TE=3.93 ms, alpha=10°, spatial resolution of 1 mm^3, two averages.

Eye tracking analysis

We used the Tracker software (https://www.cabrillo.edu/~dbrown/tracker/) to estimate the speaker's head position in the video frame by frame. The software determines the speaker's head position, in terms of coordinates x and y, in each video frame and compares it to the head position at the first frame. We then used a customized MATLAB script to correct the fixation position in accordance with the relative head positions obtained from the Tracker software. First, we used a simple formula $\frac{\sum_{i=1}^{n} x_i}{n}$ to estimate the average relative head position change (relative x and y change) within a fixation. X_i represents relative head position (either x or y) at frame i, n represents the number of frames for a given fixation. n was computed as the fixation duration divided by the frame duration (40 ms). The relative head position change (relative x and y change) was then subtracted from the raw fixation position captured by the eye tracker (raw x and y) to get

the corrected fixation position (corrected x and y). This correction was necessary, because the eye tracker captures gaze positions on the screen rather than real positions on the speaker's face. The corrected fixation positions were then used for the AOI analysis and dwell analysis described in the main text.

fMRI analyses

Analyses of BOLD response

The pre-processing procedures included slice timing, realignment and unwarp, normalization, and smoothing (full width half maximum = 6 mm). At the first-level, the general linear model (GLM) analysis for BOLD response included 6 events that had been defined by the eye tracking events (Eyes_normal, Eyes_noise, Mouth_normal, Mouth_noise, Off_normal, Off_ noise) and 7 regressors of no interest (speech recognition task, 6 spatial head movement parameters estimated during realignment). The onset of the speech recognition task regressor corresponded to the time when the question appeared on the screen. All events were modeled with a duration of 0 s. Note that the shapes and sizes of the AOIs were different (For details, see ref. 2). However, as reported in Jiang, et al. [2], the fMRI results were almost the same in the version that added the distance between sub-fixations and the number of saccades during each event as additional parameter regressors and the version that did not add these regressors. We therefore used the results without adding these regressors for further analyses.

We computed the main effect of eye contact with the Eyes vs. Mouth contrast at the first-level across the Normal and Noise conditions, i.e., (Eyes_normal + Eyes_noise)-

(Mouth_normal+Mouth_noise). We did not use the Off events for comparison, because there was a relatively low number of events and they were of relatively short duration (see IEI in Fig.S3). The contrast maps obtained at the first-level were entered into the second-level random-effects analyses using one-sample t-tests. The SPM statistical maps were thresholded at voxel-wise $p < 0.01$ with a cluster-wise family-wise error (FWE) correction of $p < 0.05$ for the whole brain. We labeled the cluster locations based on anatomical information provided by the Brodmann areas (BA) and Automated Anatomical Labeling (AAL) atlas[5] and/or probabilistic cytoarchitectonic maps[6].

Analyses of effective connectivity

The first-level PPI analysis included the physiological variable (1st eigenvariate time series from source ROI), psychological variable (Eyes vs. Mouth contrast), and interaction term (physiological × psychological variable). In addition, for the current study, we also calculated the framewise displacement (FD) for each session from the six head movement parameters obtained during realignment[7]. FD is often used to remove motion artefacts in resting-state functional connectivity analysis[7]. Therefore, we added FD as an additional regressor in the first-level analysis. This analysis was conducted for each source ROI. To examine whether the regions showed enhanced connectivity with each other during eye contact in contrast to mouth fixation, we performed ROI analyses between source ROIs and target ROIs. The target ROIs were again defined either functionally or anatomically. They were used to correct for multiple comparisons at the voxel-wise FWE of $p < 0.05$ in the second-level analyses.

Definition for source regions of interest (ROIs)

We defined source ROIs for Eigenvariate extraction in the PPI analyses based on the results in the BOLD response analyses, or if that was not possible, anatomically.

Functionally defined ROIs. For regions showed significantly higher responses to Eyes than Mouth in the whole brain, therefore we defined them functionally via 10-mm radius spheres centered on group statistical maximum coordinates (cuneus: x = -6, y = -99, z = 18 and x = 12, y = -93, z = 21; right pSTS: x = 54, y = -54, z = 15; mPFC: x = -3, y =21, z = 30; right dlPFC: x = 45, y =30, z = 36) in the Eye vs. Mouth contrast. Before extracting the 1st Eigenvariate from voxels within these spheres, we restrained only those that are above threshold (0.5) in subject-specific statistical Eye vs. Mouth contrast.

Standard anatomical maps. Standard anatomical maps were available for the bilateral amygdala (Amy), Pulvinar (Pulv), inferior temporal cortex (ITC), orbital frontal gyrus (OFC, including inferior, middle and superior parts) and lateral occipital cortex (LOC, including anterior and posterior parts) in the WFU_PickAtlas[8] or the SPM Anatomy toolbox (v2.1)[6]. Here we extracted the Eigenvariates from each of the anatomical areas for both hemispheres.

Customized anatomical masks. The fusiform gyrus (FG), the aSTS, and the superior colliculus (SC) showed no significant increases in the BOLD response analyses and no standard anatomical maps were available. We therefore made customized masks for these regions. The Fusiform Face Area (FFA) has been specifically implicated in face identity processing[9,10]. Involvement of bilateral FFA has been qualitively equally shown in

previous studies, though right side seems to have a bit larger cluster (see automated meta-analysis maps using key words 'face' in Neurosynth http://neurosynth.org/analyses/terms/face/). Moreover, previous studies with respect to eye gaze showed inconsistent finding that either bilateral FG [11] or right FG [12] for eye gaze processing. We therefore restricted the ROI of FG to bilateral FFA. We used the FSL software (Version 5.0.8, FMRIB, Oxford, UK, http://fsl.fmrib.ox.ac.uk/fsl/fslwiki/) to extract the probabilistic map of the FG and intersected it with the probabilistic atlas for face processing[13] (threshold at 0.25). Since the fast-track modulator model makes specific predictions about the role of aSTS in the right hemisphere [14], we restricted the ROI of aSTS to the right aSTS. We first extracted probabilistic maps of the temporal pole (TP) in the right hemisphere (thresholded at 0.1) with FSL software. We then restricted the TP map to the STG/S region by confining the medial boundary to the medial extent of the STS, the inferior boundary to the lower bank of the STS and the superior boundary to the lateral fissure. Because SC is a clearly visible and discrete anatomical region on a standard anatomical template, bilateral SC masks were defined as 4-mm radius spheres with reference to a brain atlas[15] using the MRIcron 3D ROI tool (http://www.mccauslandcenter.sc.edu/mricro/mricro/3droi.html). For the FFA and SC we extracted the Eigenvariates combined over both hemispheres. For aSTS we extracted the Eigenvariate from the right hemisphere.

Definition for target ROIs

The target ROIs were defined similarly to the source ROIs. For the functionally defined ROIs, we created 10-mm radius spheres around the group statistical maximum

coordinates (cuneus: $x = -6$, $y = -99$, $z = 18$ and $x = 12$, $y = -93$, $z = 21$; pSTS: $x = 54$, $y = -54$, $z = 15$; mPFC: $x = -3$, $y = 21$, $z = 30$; dlPFC: $x = 45$, $y = 30$, $z = 36$) in the Eye vs. Mouth contrast using the MRIcron 3D painting tool (http://www.mccauslandcenter.sc.edu/mricro/mricro/3droi.html). The maximum image intensity difference was set from origin to 100 and maximum brightness difference at edge to 80 to restrict the ROIs to the gray matter. All other target ROIs were identical to the source ROIs (see section *Definition for source ROIs*).

Exclusion of head movement effect

Head movement can bias functional connectivity results in people with ASD[16]. To further exclude that individual amount of head movement was not correlated with the level of autistic traits of participants in the current study, we conducted Pearson's correlation analysis. The results showed that the mean movement parameters as measured by FD were not correlated with AQ total scores ($r = -0.02$, $p = 0.924$) or the "Attention to detail" subcluster scores ($r = -0.07$, $p = 0.764$).

Mediation analysis

Sobel test

We used a Web-based interactive calculation tool, accessible at http://quantpsy.org/sobel/sobel.htm. The unstandardized regression coefficient (B) and its standard error for the correlation between the IV and MV in the MLR II and those for the

correlations between the MV and DV in the MLR III were entered into cells provided on this web page.

Results

Mediation effect revealed by Sobel test

We found the indirect effect between the IV (the FFA–pSTS effective connectivity) and DV (the "Attention to detail" subcluster scores) via the MV (the Eyes Preference) was significantly different from zero (t = -2.25, p = 0.024).

Multiple linear regression (MLR) analysis for each subscale in "Social"subcluster

The "Social"subcluster of autistic traits was not predicted by pSTS responses and FFA-pSTS connectivity. One possibility is that this was due to the slight variations between participants on 3 of 4 subscales in the social subcluster (i.e., "social skills," "communication," and "imagination," but not "attention switching"). We explored separate stepwise MLR analyses for each subscale of the "Social" subcluster, in which pSTS responses and FFA-pSTS connectivity were entered as IVs and AQ subscale scores as DV separately. We found that pSTS responses and FFA-pSTS connectivity predicted 'attention switching' scores (adjusted R^2 = 0.39, F (2, 16) = 6.73, p = 0.032 after Bonferroni correction (n=4)). The pSTS responses (β = -0.66, t = -3.44, p = 0.003) and the FFA-pSTS effective connectivity (β = -0.41, t = -2.13, p = 0.049) both showed a significant negative correlation with "attention switching" scores. There was no predictor for the other 3 subscales of the social subcluster. The mediation analysis showed no

mediation effect of Eyes preference on the relation between FFA-pSTS and “attention switching” subscale.

References

1 Schilbach, L. Eye to eye, face to face and brain to brain: novel approaches to study the behavioral dynamics and neural mechanisms of social interactions. *Curr Opin Biobehav Sci* **3**, 130-135 (2015).

2 Jiang, J., Borowiak, K., Tudge, L., Otto, C. & von Kriegstein, K. Neural mechanisms of eye contact when listening to another person talking. *Social cognitive and affective neuroscience* **12**, 319-328, doi:10.1093/scan/nsw127 (2017).

3 Vatikiotis-Bateson, E., Eigsti, I.-M., Yano, S. & Munhall, K. G. Eye movement of perceivers during audiovisualspeech perception. *Percept Psychophys* **60**, 926-940 (1998).

4 Yi, A., Wong, W. & Eizenman, M. Gaze patterns and audiovisual speech enhancement. *J Speech Lang Hear Res* **56**, 471-480, doi:10.1044/1092-4388(2012/10-0288) (2013).

5 Tzourio-Mazoyer, N. *et al.* Automated anatomical labeling of activations in SPM using a macroscopic anatomical parcellation of the MNI MRI single-subject brain. *Neuroimage* **15**, 273-289, doi:10.1006/nimg.2001.0978 (2002).

6 Eickhoff, S. B. *et al.* A new SPM toolbox for combining probabilistic cytoarchitectonic maps and functional imaging data. *Neuroimage* **25**, 1325-1335, doi:10.1016/j.neuroimage.2004.12.034 (2005).

7 Power, J. D., Barnes, K. A., Snyder, A. Z., Schlaggar, B. L. & Petersen, S. E. Spurious but systematic correlations in functional connectivity MRI networks

arise from subject motion. *Neuroimage* **59**, 2142-2154, doi:10.1016/j.neuroimage.2011.10.018 (2012).

8 Maldjian, J. A., Laurienti, P. J., Kraft, R. A. & Burdette, J. H. An automated method for neuroanatomic and cytoarchitectonic atlas-based interrogation of fMRI data sets. *Neuroimage* **19**, 1233-1239 (2003).

9 Nestor, A., Plaut, D. C. & Behrmann, M. Unraveling the distributed neural code of facial identity through spatiotemporal pattern analysis. *P Natl Acad Sci USA* **108**, 9998-10003, doi:10.1073/pnas.1102433108 (2011).

10 Rotshtein, P., Henson, R. N. A., Treves, A., Driver, J. & Dolan, R. J. Morphing Marilyn into Maggie dissociates physical and identity face representations in the brain. *Nat Neurosci* **8**, 107-113, doi:10.1038/nn1370 (2005).

11 George, N., Driver, J. & Dolan, R. J. Seen gaze-direction modulates fusiform activity and its coupling with other brain areas during face processing. *Neuroimage* **13**, 1102-1112 (2001).

12 Calder, A. J. *et al.* Reading the mind from eye gaze. *Neuropsychologia* **40**, 1129-1138 (2002).

13 Engell, A. D. & McCarthy, G. Probabilistic atlases for face and biological motion perception: An analysis of their reliability and overlap. *Neuroimage* **74**, 140-151, doi:10.1016/j.neuroimage.2013.02.025 (2013).

14 Senju, A. & Johnson, M. H. The eye contact effect: mechanisms and development. *Trends Cogn Sci* **13**, 127-134, doi:10.1016/j.tics.2008.11.009 (2009).

15 Duvernoy, H. (New York: Springer-Verlag, 1991).

16 Deen, B. & Pelphrey, K. Perspective: Brain scans need a rethink. *Nature* **491**, S20 (2012).

Supplementary Figures

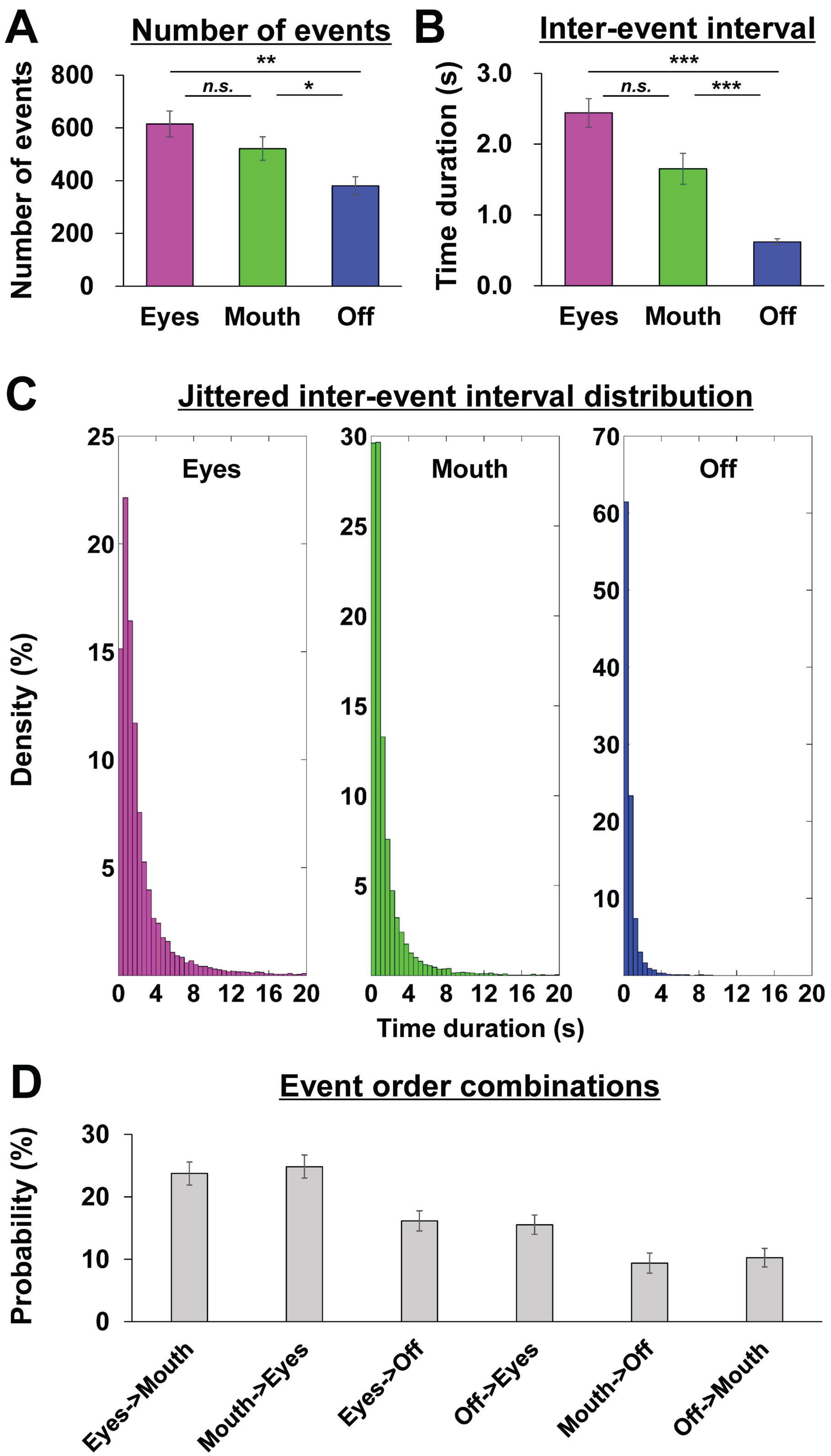
A
Number of events
**
n.s.
*
800
600
400
200
0
Number of events
Eyes
Mouth
Off
B
Inter-event interval
***
n.s.
***
3.0
2.0
1.0
0.0
Time duration (s)
Eyes
Mouth
Off
C
Jittered inter-event interval distribution
Density (%)
Eyes
Mouth
Off
0 4 8 12 16 20
Time duration (s)
D
Event order combinations
Probability (%)
30
20
10
0
Eyes->Mouth
Mouth->Eyes
Eyes->Off
Off->Eyes
Mouth->Off
Off->Mouth

Figure S1. Eye gaze patterns during free viewing of the monologue videos pooled for the Normal and Noise conditions. ***A-B,*** The number of events and inter-event interval. The inter-event interval is defined by the time interval between the onset of one event and that of the next one. Error bars display the standard error of the mean. ***C,*** Density histograms indicating inter-event interval distribution for Eyes, Mouth and Off. Eyes, Mouth and Off represent fixations falling into eye region, mouth region and outside of these regions, respectively. ***D,*** Probability of all possible combinations of event orders, that is, Eyes->Mouth means the fixation shifts from eye region to mouth region. Figure adapted from Jiang, et al. [2].

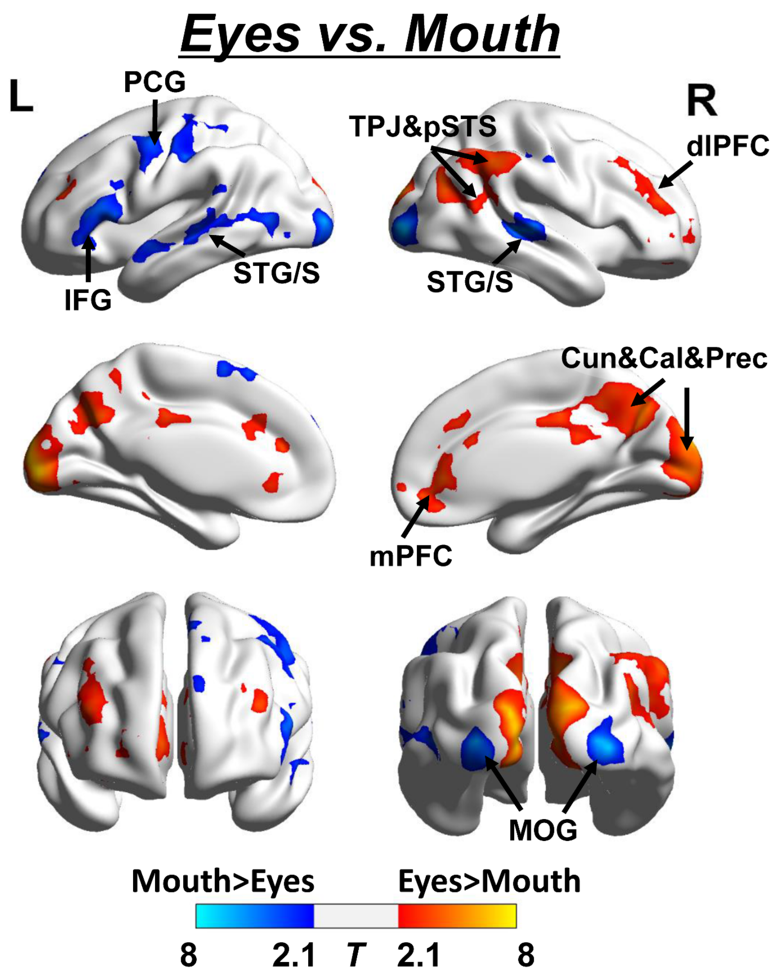

Figure S2. Brain regions specifically responsive to eye contact and mouth fixation. Hot colors (red to yellow) indicate brain areas showing higher response to Eye contact than mouth fixation. Cold colors (blue to cyan) showed higher response to Mouth fixations than eye contact. L, left hemisphere, R, right hemisphere. For visualization purposes only, voxels that survived with a voxel-level threshold of $p < 0.05$ and 160 voxels are shown. Cun, cuneus; Cal, calcarine; Prec, precuneus; TPJ, temporoparietal junction; STS, superior temporal sulcus; mPFC, medial prefrontal cortex; dlPFC, dorsolateral prefrontal cortex; MOG, middle occipital cortex; IFG, inferior frontal gyrus; PCG, precentral and/or postcentral gyrus. Figure adapted from Jiang, et al. [2]

A

Target region (columns) / **Source region** (rows)

	bCun	bSC	bAmy	bPulv	bITC	bLOC	rdlPFC	bOFC	rpSTS	lmPFC	raSTS	bFFA
rCun		0.206	0.095	0.048	0.130	0.013	0.062	0.111	0.012	0.047	0.119	0.027
bSC	0.602		0.893	0.145	0.847	0.332	0.819	0.384	0.638	0.772	0.064	0.664
bAmy	0.293	0.056		0.617	0.129	0.708	0.759	0.715	0.129	0.129	0.325	0.563
bPulv	0.040	0.117	0.404		0.129	0.337	0.416	0.361	0.149	0.061	0.068	0.348
bITC	0.637	0.029	0.218	0.539		0.342	0.516	0.212	0.027	0.671	0.059	0.034
bLOC	0.558	0.210	0.023	0.073	0.264		0.541	0.017	0.017	0.059	0.038	0.053
rdlPFC	0.181	0.357	0.525	0.367	0.361	0.078		0.228	0.009	0.119	0.267	0.121
bOFC	0.249	0.120	0.511	0.378	0.581	0.475	0.046		0.077	0.011	0.308	0.640
rpSTS	0.741	0.318	0.134	0.782	0.612	0.228	0.800	0.688		0.476	0.275	0.127
lmPFC	0.634	0.065	0.671	0.642	0.806	0.363	0.460	0.318	0.234		0.453	0.348
raSTS	0.478	0.326	0.805	0.282	0.242	0.387	0.601	0.090	0.220	0.170		0.050
bFFA	0.168	0.084	0.001	0.129	0.220	0.018	0.692	0.156	0.010	0.427	0.025	

$p < 0.05$, FWE corrected (yellow)   **$p < 0.06$, FWE corrected** (orange)

B

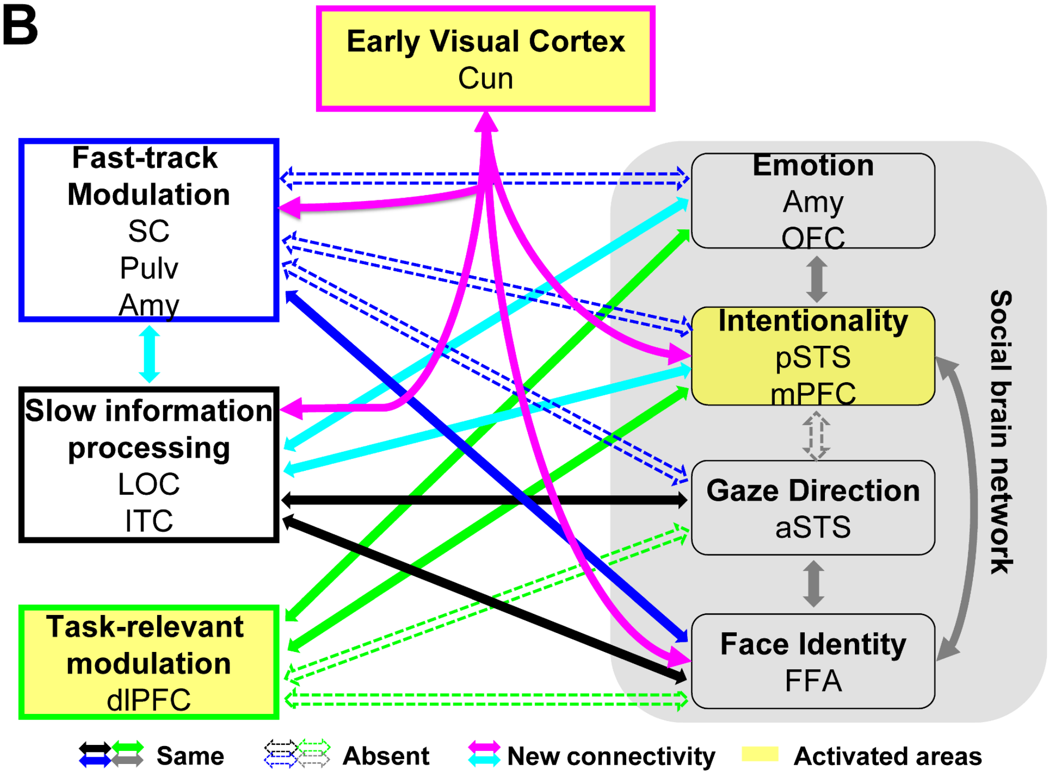

Figure S3. Effective connectivity results revealed by ROI-based PPI analyses after removing effect of head movement. ***A,*** All values represent FWE corrected *p* values for enhanced connectivity between source regions (for physiological variable extraction) and target regions (for small volume correction) for eye contact in contrast to mouth fixation. Values marked with yellow are significant at $p < 0.05$, voxel-wise FWE corrected. Values in orange are present at $p < 0.06$ and values in light blue display corrected *p* values for non-significant connectivity. Note that because the connectivity findings for right and left cuneus as source region were qualitatively similar, only the findings of the right cuneus are displayed here. ***B,*** Comparison of effective connectivity found in the current study and that predicted in the fast-track modulator model[14] (also see Analyses of effective connectivity). Solid arrows represent connectivity found in the PPI analyses that were predicted by the fast-track modulator model. Dashed arrows represent connectivity predicted in the model, but not found in our results. Pink and cyan solid arrows represent new connectivity found in the current study, but not predicted by the model. Brain areas marked with yellow are the areas found to be significantly more responsive to eye contact vs. mouth fixation in the GLM analysis (see also Fig. S2). b, bilateral; r, right; l, left. Cun, Cuneus; SC, superior colliculus; Pulv, pulvinar; LOC, lateral occipital cortex; OFC, orbitofrontal cortex; mPFC, medial prefrontal cortex; FFA, fusiform face area; dlPFC, dorsolateral prefrontal cortex; Amy, amygdala; a/pSTS, anterior/posterior superior temporal sulcus; ITC, inferior temporal cortex.

Supplementary Tables

Table S1. The Autistic-Spectrum Quotient

1. I prefer to do things with others rather than on my own.	definitely agree	slightly agree	slightly disagree	definitely disagree
2. I prefer to do things the same way over and over again.	definitely agree	slightly agree	slightly disagree	definitely disagree
3. If I try to imagine something, I find it very easy to create a picture in my mind.	definitely agree	slightly agree	slightly disagree	definitely disagree
4. I frequently get so strongly absorbed in one thing that I lose sight of other things.	definitely agree	slightly agree	slightly disagree	definitely disagree
5. I often notice small sounds when others do not.	definitely agree	slightly agree	slightly disagree	definitely disagree
6. I usually notice car number plates or similar strings of information.	definitely agree	slightly agree	slightly disagree	definitely disagree
7. Other people frequently tell me that what I've said is impolite, even though I think it is polite.	definitely agree	slightly agree	slightly disagree	definitely disagree
8. When I'm reading a story, I can easily imagine what the characters might look like.	definitely agree	slightly agree	slightly disagree	definitely disagree
9. I am fascinated by dates.	definitely agree	slightly agree	slightly disagree	definitely disagree
10. In a social group, I can easily keep track of several different people's conversations.	definitely agree	slightly agree	slightly disagree	definitely disagree
11. I find social situations easy.	definitely agree	slightly agree	slightly disagree	definitely disagree
12. I tend to notice details that others do not.	definitely agree	slightly agree	slightly disagree	definitely disagree
13. I would rather go to a library than a party.	definitely agree	slightly agree	slightly disagree	definitely disagree
14. I find making up stories easy.	definitely agree	slightly agree	slightly disagree	definitely disagree
15. I find myself drawn more strongly to people than to things.	definitely agree	slightly agree	slightly disagree	definitely disagree
16. I tend to have very strong interests, which I get upset about if I can't pursue.	definitely agree	slightly agree	slightly disagree	definitely disagree
17. I enjoy social chit-chat.	definitely agree	slightly agree	slightly disagree	definitely disagree

18. When I talk, it isn't always easy for others to get a word in edgeways.	definitely agree	slightly agree	slightly disagree	definitely disagree
19. I am fascinated by numbers.	definitely agree	slightly agree	slightly disagree	definitely disagree
20. When I'm reading a story, I find it difficult to work out the characters' intentions.	definitely agree	slightly agree	slightly disagree	definitely disagree
21. I don't particularly enjoy reading fiction.	definitely agree	slightly agree	slightly disagree	definitely disagree
22. I find it hard to make new friends.	definitely agree	slightly agree	slightly disagree	definitely disagree
23. I notice patterns in things all the time.	definitely agree	slightly agree	slightly disagree	definitely disagree
24. I would rather go to the theatre than a museum.	definitely agree	slightly agree	slightly disagree	definitely disagree
25. It does not upset me if my daily routine is disturbed.	definitely agree	slightly agree	slightly disagree	definitely disagree
26. I frequently find that I don't know how to keep a conversation going.	definitely agree	slightly agree	slightly disagree	definitely disagree
27. I find it easy to "read between the lines" when someone is talking to me.	definitely agree	slightly agree	slightly disagree	definitely disagree
28. I usually concentrate more on the whole picture, rather than the small details.	definitely agree	slightly agree	slightly disagree	definitely disagree
29. I am not very good at remembering phone numbers.	definitely agree	slightly agree	slightly disagree	definitely disagree
30. I don't usually notice small changes in a situation, or a person's appearance.	definitely agree	slightly agree	slightly disagree	definitely disagree
31. I know how to tell if someone listening to me is getting bored.	definitely agree	slightly agree	slightly disagree	definitely disagree
32. I find it easy to do more than one thing at once.	definitely agree	slightly agree	slightly disagree	definitely disagree
33. When I talk on the phone, I'm not sure when it's my turn to speak.	definitely agree	slightly agree	slightly disagree	definitely disagree
34. I enjoy doing things spontaneously.	definitely agree	slightly agree	slightly disagree	definitely disagree
35. I am often the last to understand the point of a joke.	definitely agree	slightly agree	slightly disagree	definitely disagree
36. I find it easy to work out what someone is	definitely	slightly	slightly	definitely

thinking or feeling just by looking at their face.	agree	agree	disagree	disagree
37. If there is an interruption, I can switch back to what I was doing very quickly.	definitely agree	slightly agree	slightly disagree	definitely disagree
38. I am good at social chit-chat.	definitely agree	slightly agree	slightly disagree	definitely disagree
39. People often tell me that I keep going on and on about the same thing.	definitely agree	slightly agree	slightly disagree	definitely disagree
40. When I was young, I used to enjoy playing games involving pretending with other children.	definitely agree	slightly agree	slightly disagree	definitely disagree
41. I like to collect information about categories of things (e.g. types of car, types of bird, types of train, types of plant, etc.).	definitely agree	slightly agree	slightly disagree	definitely disagree
42. I find it difficult to imagine what it would be like to be someone else.	definitely agree	slightly agree	slightly disagree	definitely disagree
43. I like to plan any activities I participate in carefully.	definitely agree	slightly agree	slightly disagree	definitely disagree
44. I enjoy social occasions.	definitely agree	slightly agree	slightly disagree	definitely disagree
45. I find it difficult to work out people's intentions.	definitely agree	slightly agree	slightly disagree	definitely disagree
46. New situations make me anxious.	definitely agree	slightly agree	slightly disagree	definitely disagree
47. I enjoy meeting new people.	definitely agree	slightly agree	slightly disagree	definitely disagree
48. I am a good diplomat.	definitely agree	slightly agree	slightly disagree	definitely disagree
49. I am not very good at remembering people's date of birth.	definitely agree	slightly agree	slightly disagree	definitely disagree
50. I find it very easy to play games with children that involve pretending	definitely agree	slightly agree	slightly disagree	definitely disagree

Table S2. Number of events across conditions and within condition for each participant

Subjects	Across Conditions			Normal Condition			Noise Condition			Total
	Eyes	Mouth	Off	Eyes	Mouth	Off	Eyes	Mouth	Off	
sub01	407	808	629	200	355	265	207	453	364	1844
sub02	517	467	355	283	231	202	234	236	153	1339
sub03	499	600	594	301	302	308	198	298	286	1693
sub04	848	674	290	340	224	129	508	450	161	1812
sub05	435	399	227	222	201	114	213	198	113	1061
sub06	659	535	370	317	252	205	342	283	165	1564
sub07	886	501	556	355	161	239	531	340	317	1943
sub08	881	523	461	462	236	269	419	287	192	1865
sub09	505	562	348	299	316	204	206	246	144	1415
sub10	445	465	243	283	279	156	162	186	87	1153
sub11	521	551	349	352	349	229	169	202	120	1421
sub12	551	287	428	258	106	191	293	181	237	1266
sub13	838	625	317	410	264	198	428	361	119	1780
sub14	1011	915	197	468	416	95	543	499	102	2123
sub15	275	210	90	130	85	38	145	125	52	575
sub16	528	196	459	257	95	233	271	101	226	1183
sub17	920	771	459	431	354	193	489	417	266	2150
sub18	416	288	249	210	131	124	206	157	125	953
sub19	541	526	606	292	217	278	249	309	328	1673
Average	614.89	521.21	380.37	308.95	240.74	193.16	305.95	280.47	187.21	1516.47
SD	213.55	193.81	149.61	90.42	95.59	69.48	136.50	116.11	90.14	419.19

Table S3. Inter-event interval across conditions and within condition for each participant

Subjects	Across Conditions			Normal Condition			Noise Condition		
	Eyes	Mouth	Off	Eyes	Mouth	Off	Eyes	Mouth	Off
sub01	1.62	1.68	1.08	2.11	1.72	1.09	1.15	1.65	1.06
sub02	2.53	2.31	0.74	2.82	1.76	0.77	2.19	2.86	0.70
sub03	1.31	2.48	0.81	1.48	2.05	0.72	1.05	2.92	0.91
sub04	2.27	0.83	0.69	3.37	0.64	0.61	1.53	0.93	0.75
sub05	2.34	2.07	0.64	2.38	1.93	0.58	2.31	2.22	0.71
sub06	2.58	1.33	0.73	2.79	1.18	0.61	2.39	1.47	0.88
sub07	2.54	0.53	0.32	3.24	0.50	0.34	2.07	0.54	0.31
sub08	2.32	0.76	0.53	2.40	0.71	0.51	2.25	0.80	0.56
sub09	1.76	2.87	0.55	1.95	1.86	0.52	1.47	4.17	0.59
sub10	2.00	3.42	0.83	1.99	2.14	0.71	2.02	5.33	1.03
sub11	1.19	2.86	0.58	1.24	2.03	0.60	1.09	4.31	0.54
sub12	3.78	0.56	0.42	4.53	0.44	0.34	3.13	0.62	0.48
sub13	2.52	0.72	0.41	2.80	0.67	0.45	2.26	0.76	0.34
sub14	1.84	0.81	0.46	2.17	0.76	0.41	1.55	0.86	0.51
sub15	7.38	2.98	0.39	9.95	0.70	0.42	5.07	4.53	0.36
sub16	4.25	0.73	0.63	4.49	0.71	0.62	4.02	0.76	0.64
sub17	1.93	0.79	0.60	2.03	0.80	0.71	1.85	0.78	0.52
sub18	4.48	1.80	0.74	5.03	1.73	0.77	3.92	1.86	0.71
sub19	2.42	1.83	0.67	2.93	1.55	0.65	1.82	2.02	0.69
Average	2.69	1.65	0.62	3.14	1.26	0.60	2.27	2.07	0.65
SD	1.44	0.95	0.18	1.94	0.62	0.18	1.07	1.53	0.22
